# Coral and Seawater Metagenomes Reveal Key Microbial Functions to Coral Health and Ecosystem Functioning Shaped at Reef Scale

**DOI:** 10.1007/s00248-022-02094-6

**Published:** 2022-08-15

**Authors:** Laís F. O. Lima, Amanda T. Alker, Bhavya Papudeshi, Megan M. Morris, Robert A. Edwards, Samantha J. de Putron, Elizabeth A. Dinsdale

**Affiliations:** 1 Department of Biology, San Diego State University, San Diego, CA USA; 2 College of Biological Sciences, University of California Davis, Davis, CA USA; 3 College of Science and Engineering, Flinders University, Adelaide, South Australia Australia; 4 Lawrence Livermore National Laboratory, Livermore, CA USA; 5 Bermuda Institute of Ocean Sciences, St. George’s, Bermuda

**Keywords:** Host-Microbiome, Acclimatization, Resilience, Environmental Change, Coral Reefs

## Abstract

The coral holobiont is comprised of a highly diverse microbial community that provides key services to corals such as protection against pathogens and nutrient cycling. The coral surface mucus layer (SML) microbiome is very sensitive to external changes, as it constitutes the direct interface between the coral host and the environment. Here, we investigate whether the bacterial taxonomic and functional profiles in the coral SML are shaped by the local reef zone and explore their role in coral health and ecosystem functioning. The analysis was conducted using metagenomes and metagenome-assembled genomes (MAGs) associated with the coral *Pseudodiploria strigosa* and the water column from two naturally distinct reef environments in Bermuda: inner patch reefs exposed to a fluctuating thermal regime and the more stable outer reefs. The microbial community structure in the coral SML varied according to the local environment, both at taxonomic and functional levels. The coral SML microbiome from inner reefs provides more gene functions that are involved in nutrient cycling (e.g., photosynthesis, phosphorus metabolism, sulfur assimilation) and those that are related to higher levels of microbial activity, competition, and stress response. In contrast, the coral SML microbiome from outer reefs contained genes indicative of a carbohydrate-rich mucus composition found in corals exposed to less stressful temperatures and showed high proportions of microbial gene functions that play a potential role in coral disease, such as degradation of lignin-derived compounds and sulfur oxidation. The fluctuating environment in the inner patch reefs of Bermuda could be driving a more beneficial coral SML microbiome, potentially increasing holobiont resilience to environmental changes and disease.

**Supplementary Information:**

The online version contains supplementary material available at 10.1007/s00248-022-02094-6.

## Introduction

Reef-building corals are considered model organisms to study host-associated microbiomes under environmental changes [1, 2]. Coral colonies function as a holobiont in which the coral animal associates with endosymbiotic dinoflagellates of the family *Symbiodiniaceae* and a diverse community of bacteria, archaea, fungi, and viruses [3]. The coral holobiont depends on nutrient cycling (e.g., nitrogen and sulfur cycling) mediated by the associated microbiome [4–7]. The coral surface mucous layer (SML) sustains a high abundance (10^6^–10^8^ cells per milliliter) and diversity of these microbial partners [8–11]. Corals invest up to 50% of fixed carbon on mucus production [12, 13] for physical protection and to trap organic matter that can be consumed via heterotrophy [14, 15]. The coral mucus and associated microbial community influences nutrient fluxes into the benthos, water column, and sediment [15–20] thus shaping the ecosystem functions. The coral microbiome benefits from the high nitrogen content and organic matter in the SML [7, 17] and provides protection against coral pathogens via production of antimicrobials [21, 22]. However, coral-associated microbial communities are sensitive to environmental changes, particularly to increased temperature and nutrient concentration, which disrupt the beneficial services provided to the holobiont [23–26]. Therefore, the coral SML microbiome constitutes a direct interface between the coral host and the environment and is strongly influenced by the microbial community in the water column [27, 28].

The acclimatization mechanisms of the coral holobiont to changing environmental conditions are not completely understood; however, the coral microbiome is recognized as a major player. The microbial-mediated transgenerational acclimatization (MMTA) theory hypothesizes that the coral holobiont benefits from inheritable microbial taxa and/or genes acquired and/or selected in the coral microbiome when exposed to environmental changes [29]. Within the coral microbiome, there is a diversity of microbial taxa with traits that potentially improve coral fitness and resilience [30]. For example, the associated microbial community is a potential source of acquired heat tolerance [31]. Corals develop resilience to stress factors by associating with certain microorganisms and maintaining their “health-state” microbial taxonomic composition under stress or rapidly recovering to the “health-state” microbes after disturbances [32]. Microbial functional profiles also respond to environmental gradients and can be used to identify changes in host health and ecosystem functioning [33–35]. Determining which microbial taxa and functional genes are available in the surrounding environment and how they are being selected in the coral microbiome is key to provide a foundation to theories such as MMTA applied to the coral holobiont.

Coral reef microbial ecology has benefited from the advancement of shotgun metagenomics to provide an in-depth description of the microbial taxa and functional genes that play a key role in the health of reef ecosystems [24, 36–39]. Shotgun metagenomics is not restricted to marker genes such as 16S rRNA in amplicon metagenomics, which results in a more complete profile of the microbial taxa and metabolic potential of functional genes [40, 41]. However, the use of shotgun metagenomics in coral reef microbiology has traditionally focused on sequencing the microbial communities in reef water [25, 35, 37, 42–46]. Consequently, the microbial functional profile in the coral holobiont is still underexplored [47]. Here, we investigate whether the microbial taxonomic and functional profiles in the coral SML are shaped by their local reef environment and explore their role in coral health and ecosystem functioning.

## Methods

### Aim of the Study

We compared the metagenomes associated with the brain coral *Pseudodiploria strigosa* (Dana, 1846) and the water column sampled in situ from two naturally distinct reef environments in Bermuda. The reef system in Bermuda is the most northern in the Atlantic and experiences large seasonal variations in environmental conditions [48]. In addition, fine-scale variations in temperature, light, and seawater chemistry occur between the outer rim reefs at the edge of the platform and inner lagoon patch reefs [49] with the inner patch reefs historically being warmer and more thermally variable [48, 50–53]. We showed in Lima et al. [54] that the coral SML microbiome from the inner patch reefs and the outer rim reefs in Bermuda can be modeled according to the local annual thermal profile. Here, we expand the analysis to a fine-scale taxonomic level (i.e., microbial genera and metagenome assembled genomes—MAGs) and to the functional level (i.e., SEED subsystems and pathways) in the microbial communities from the coral SML and surrounding water across these reef zones.

### In Situ Collections

We selected *P. strigosa* as the coral host species because it is widely distributed across the Bermuda platform. The reef zones sampled were approximately 8 km apart [54] and *P. strigosa* is a broadcast spawner; therefore, there is a high likelihood that gene flow between the coral hosts colonizing inner and outer reefs is maintained and that the host genetics is not structured into different populations. Indeed, studies on other species have indicated high genetic exchange among reef sites in Bermuda [55, 56]. The sampling period occurred between May 18th and May 22nd, 2017, late spring in the northern hemisphere, when environmental conditions between the two reef zones, especially temperature, are similar. The environmental gradient assessed here are based on the knowledge that these two reef zones are exposed to different regimes on a seasonal basis, with the most striking fluctuations occurring in the winter and summer months [48–50]. Therefore, we selected this period to capture a potential long-term acclimatization of the coral holobiont to their reef zones, and not their immediate response to acute temperature fluctuations. Each reef zone was replicated across three reef sites [54]. The SML of *P. strigosa* was collected from six colonies (diameter, 10 to 15 cm) from the inner and outer reef zones (*n* = 12 colonies total) using a modified two-way 50-ml syringe filled with 0.02-µm-filtered seawater [54] that dislodges the microbes and recollects the microbial-mucus slurry in the backside of the syringe. We collected 200 ml of coral mucus-microbe slurry (four syringes applied to different parts of the colony’s surface) per colony to increase DNA concentration per sample. The reef water (volume = 10 l per replicate) was collected about 1 m above the coral colonies from the inner and outer reef zones (*n* = 12 replicates total). Coral SML and water samples were pushed through a 0.22-µm Sterivex filter (EMD Millipore) for DNA extraction. The collections were performed via SCUBA diving at a depth of 4 to 6 m. A Manta2 Series MultiprobeTM was used to measure pH (0–14 units), water temperature (°C), chlorophyll concentrations (µg/l), and dissolved oxygen (% saturation and mg/l) across a 6-m depth profile at each sampling site. Our benthic survey methods were based on the Atlantic and Gulf Rapid Reef Assessment (AGRRA) Program protocols [57]. The benthic cover was measured via 10-m line transects (*n* = 3 per site) using the point intercept method every 10 cm (100 points total). Corals were identified at species level and the other organisms categorized in the following groups: macroalgae, turf algae, crustose coralline algae, gorgonian, milleporid, sponge, and other.

### Metagenomic Analysis

Microbial DNA from the coral mucus and seawater collected on the 0.22-µm Sterivex was extracted using a modified Macherey–Nagel protocol using NucleoSpin column for purification. DNA was stored at − 20 °C until quantification with Qubit (Thermo Fisher Scientific) [37]. The Swift kit 2S plus (Swift Biosciences) was used for library preparation since it provides good results from small amounts of input DNA, characteristic of microbial samples collected from the surface of the host [58, 59]. All samples were sequenced by the Dinsdale lab on Illumina MiSeq at San Diego State University. The sequenced DNA was analyzed for quality control using PrinSeq [60] before annotation. The metagenomes were annotated through MG-RAST [61], using the RefSeq database for taxonomic annotations and the SEED database for functional annotations. The number of sequence hits for each microbial taxon or function is represented as the relative abundance by calculating the proportion of sequence hits for that parameter over the total number of sequences annotated for that metagenome. Metagenomes were compared using proportional abundance, which is preferred to rarefaction [62–64]. We used metagenomics to describe the abundance of genes in the microbiome as a proxy for gene expression: although it does not measure which functional genes are being expressed at the point the sample was taken, it measures which functional genes are important for the microbes in that environment [65, 66]. There is a high level of correlation between the metagenomes and metatranscriptomes [67], where the abundance of a gene in metagenomes is a predictor of its expression level in the metatranscriptome and areas where the two analyses vary are associated with short-term changes in expression rather than bacteria functions that are under strong selective pressure and are well adapted to their environment [68–70].

### Metagenome-Assembled Genomes

MAGs were constructed to identify the level of shared taxa between the coral SML from the two locations. All the coral SML metagenomes post-quality control using Prinseq [60] were cross-assembled using megahit [71] and spades [72]. To remove the redundancy in the assembled contigs, bbtools program [73] dedupe.sh script was to remove 15% of contigs that were exact duplicates. The resulting contigs were run through Metabat2 [74] and CONCOCT [75] binning tools to generate 38 MAGs and 167 MAGs, respectively. DasTool [76] was run on these bins to generate 82 non-redundant set of MAGs. CheckM [77] was run on these 82 MAGs to assess the completeness and contamination within each MAGs. The MAGs were annotated through PATRIC version 3.6.9 using RAST tool kit (RASTtk) [78]. MAGs were described following the minimum standards for MAGs [47, 79].

### Statistical Analysis

Statistical analyses were conducted using PRIMER v7 plus PERMANOVA, Statistical Analyses of Metagenomic Profiles (STAMP) software [80], and R (R Project for Statistical Computing). Significant differences in the relative abundances of microbial genera and functions in the coral microbial communities sampled from inner and outer reefs were identified by permutational multivariate analysis of variance (PERMANOVA) using Bray–Curtis distances of normalized relative abundance obtained using a fourth-root transformation. The fourth-root transformation balances the effects of a community structured on a few abundant species and a community structured on all species, and thereby influenced by the occurrence of the rarest taxa [81, 82]. A principal coordinate analysis was created to visualize the separation of the coral microbiome between inner and outer reefs. We also used PRIMER to calculate Pielou’s evenness index (J’) and Shannon’s diversity index (H’) of microbial genera. The multiple comparisons of either taxa or functions across the four groups of metagenomes (i.e., outer coral, outer water, inner coral, and inner water) were conducted in STAMP using ANOVA/Tukey–Kramer and Benjamini–Hochberg FDR corrections. We used R to test parametric assumptions of normality (Shapiro–Wilk’s test) and homoscedasticity (Bartlett’s test), and pairwise comparisons between relative abundances of gene pathways (Student’s *T*-test).

## Results

### Taxonomic Profile

The metagenomes associated with the coral SML of *P. strigosa* and the water column sampled from inner and outer reefs in Bermuda (*n* = 24) were sequenced at high coverage, ranging from 421,976 to 1,368,678 sequence counts. Bacteria accounted for approximately 99% of the annotation (Table S1); therefore, we are only analyzing bacterial taxa and gene functions in this study. The metagenomes were assigned to four different groups (total *n* = 24 with 6 metagenomes in each group) according to their host medium and location: inner reef corals, inner reef water, outer reef corals, and outer reef water. Microbial richness did not vary significantly between groups or samples, ranging from 581 to 587 bacterial genera identified, including 23 taxa unclassified at genus level, across all metagenomes. Evenness (J’) of bacterial genera was slightly lower in inner reefs (coral 0.72 ± 0.03, water 0.72 ± 0.02) when compared to outer reefs (coral 0.75 ± 0.01, water 0.75 ± 0.01), which translated in a higher diversity index (H’) in outer reef samples (coral 4.78 ± 0.06, water 4.80 ± 0.08) than in inner reef samples (coral 4.56 ± 0.17, water 4.59 ± 0.11).

**Fig. 1:**
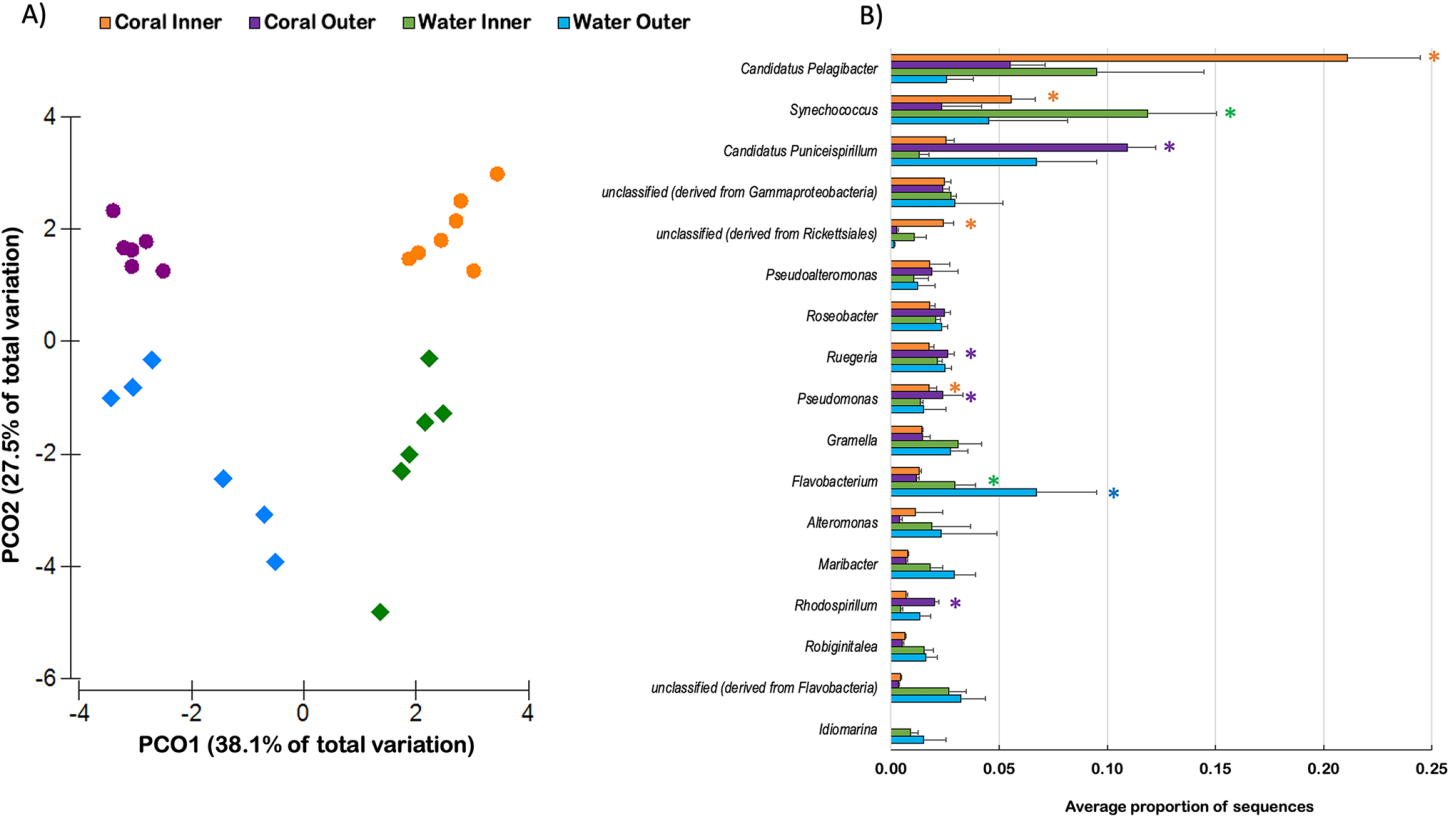
Clear differences in taxonomic make-up of the microbial community are shown using a principal coordinate analysis (**A**) based on a Bray–Curtis similarity matrix of the relative abundance of bacterial genera associated with the SML microbiome of corals (circles) and the water column (diamonds) from inner and outer reefs. Bacterial genera (mean ± SD; average abundances > 1%) showed significantly different proportions (**B**) according to multiple comparison Tukey–Kramer tests (asterisks indicate *p* < 0.05)

In contrast to diversity metrics, the microbial community structure (i.e., relative abundance of taxa) was significantly different between the four groups (PERMANOVA, pseudo-*F* = 10.8, *p* < 0.001). The metagenomes clustered according to the reef zone and were more similar to one another among the coral-associated samples than the water samples (Fig. 1A). Among the most abundant taxa (i.e., average relative abundance > 1% in a least one of the four groups), eight bacterial genera were significantly overrepresented according to their associated environment (Fig. 1B). The SML microbiome of corals from the inner reef zone had a greater relative abundance of the alphaproteobacterium Candidatus *Pelagibacter*, and of an unclassified genus, also belonging to the order *Rickettsiales*, compared to all other groups (ANOVA, Eta-squared = 0.93, *p* < 0.001). The relative abundance of cyanobacterium *Synechococcus* (ANOVA, Eta-squared = 0.62, *p* < 0.001) was greater in the water microbiome from inner reefs compared to the microbiome from both water and coral in outer reefs (Tukey–Kramer, *p* < 0.01). This overrepresentation was reflected in the coral SML microbiome from inner reefs compared to the coral SML microbiome from outer reefs (*p* < 0.05). The SML microbiome of corals from outer reefs showed a greater abundance of alphaproteobacteria Candidatus *Puniceispirillum* (ANOVA, Eta-squared = 0.92, Tukey–Kramer, *p* < 0.001), *Ruegeria* (ANOVA, Eta-squared = 0.73, *p* < 0.001), and *Rhodospirillum* (ANOVA, Eta-squared = 0.92, Tukey–Kramer, *p* < 0.001) compared to all groups. The coral SML microbiomes from both reef zones were enriched with gammaproteobacteria of the genus *Pseudomonas* (ANOVA, Eta-squared = 0.61, *p* < 0.001) when compared to the surrounding water microbiome from their respective local environment (Tukey–Kramer, *p* < 0.05). In contrast, *Flavobacterium* had a greater representation in the microbial communities from the water of both reef environments than in the microbiome associated with corals from inner and outer reefs (ANOVA, Eta-squared = 0.61, Tukey–Kramer, *p* < 0.01).

**Fig. 2:**
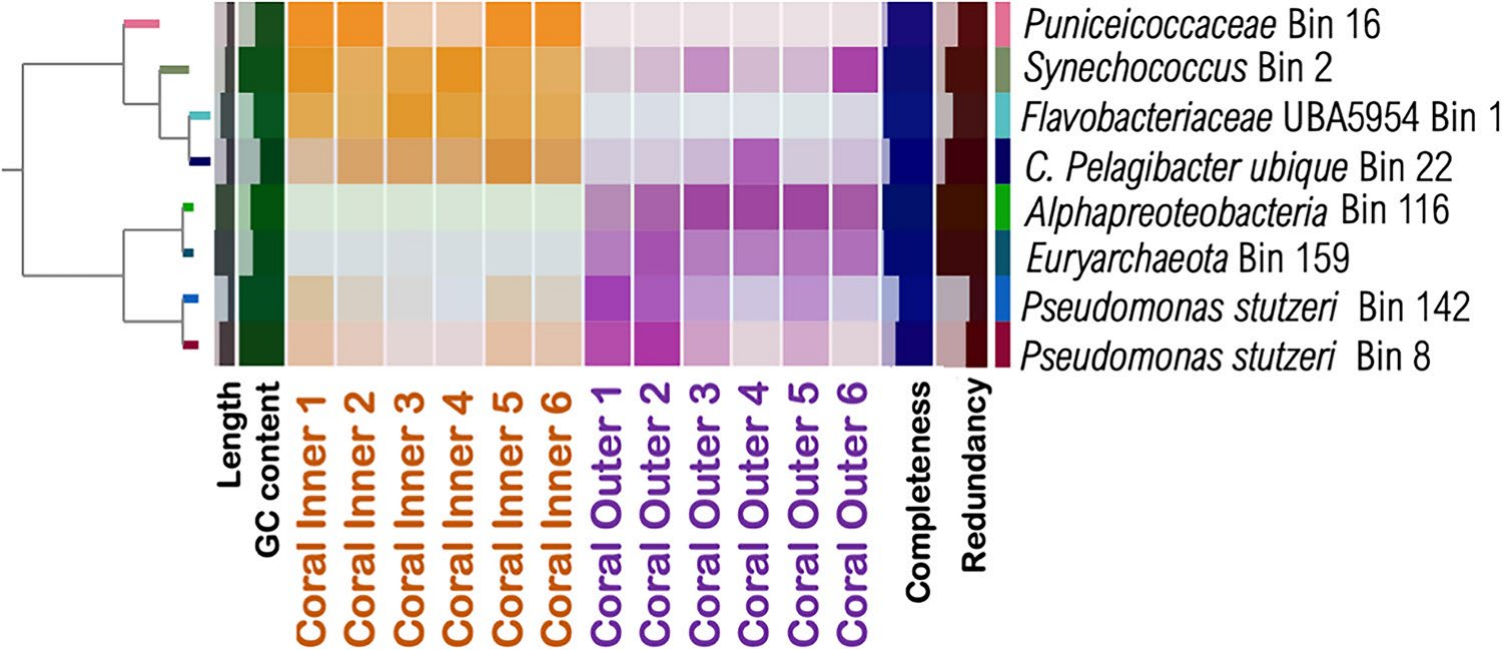
Metagenome-assembled genomes (MAGs) of eight bins generated from the twelve coral SML metagenomic samples. The heatmap shows the contribution of each metagenome to the formation of each individual bin; organized by hierarchical clustering tree using Euclidean distance and Ward linkage

MAGs indicated a clear separation between the coral SML microbiome from inner and outer reefs (Fig. 2). A total of 82 bins were constructed, and we selected eight MAGs with high levels of completeness (53 < 98%) and that represented a wide range of taxonomic variation according to the preliminary annotation from CheckM for further analysis. A hierarchical clustering tree separated the bins into two major clusters, each with four MAGs, including bacterial and archaeal taxa. The first cluster was formed by MAGs annotated as *Puniceicoccaceae* (Bin 16), *Synechoccocus* (Bin 2), *Flavobacteriaceae* (Bin 1), and Candidatus *Pelagibacter ubique* (Bin 22). The metagenomes that contributed to most to the bins in this cluster were samples from the SML of inner reef corals. The second cluster was comprised of MAGs annotated as *Alphaproteobacteria* (Bin 116), *Euryarchaeota* (Bin 159), and *Pseudomonas stutzeri* (Bin 8 and Bin 142). The metagenomes that contributed to each of the MAGs in this cluster were majorly samples from the SML of outer reef corals.

### Functional Profile

**Fig. 3:**
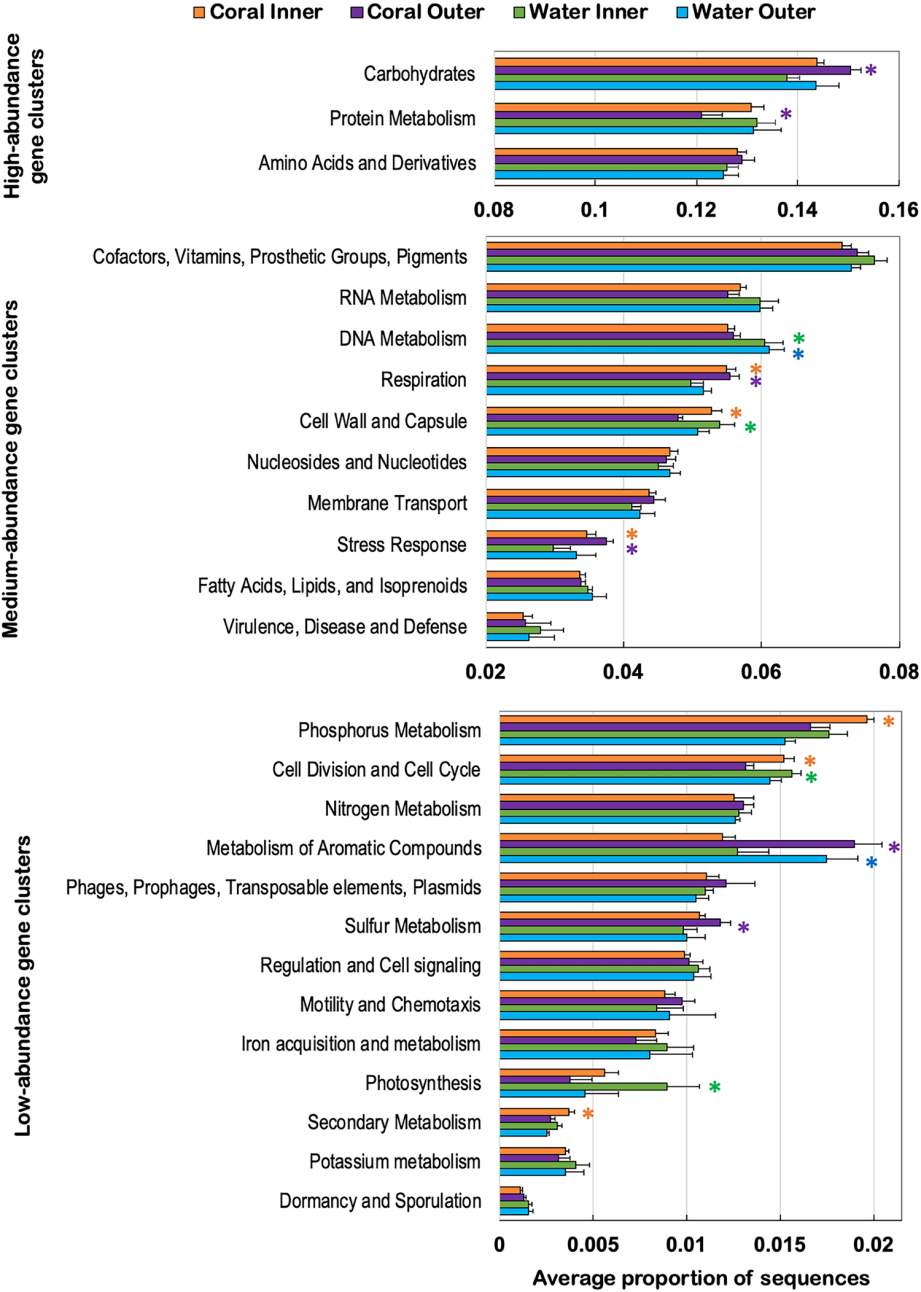
Bacterial broad functional gene categories (SEED subsystem 1) (mean ± SD associated with the SML microbiome of corals, and the water column from inner and outer reefs showed significantly different proportions according to multiple comparison Tukey–Kramer tests (asterisks indicate *p* < 0.05)

The microbial communities associated with the coral SML and water column from inner and outer reefs revealed specific functional traits. Bacterial genes classified at the broadest functional categories (SEED subsystem level 1) significantly varied across the four groups (PERMANOVA, pseudo-*F* = 8.49, *p* < 0.001). From a total of 26 broad functional categories, 12 were significantly overrepresented according to their associated environment (Fig. 3). The microbiome of corals from outer reefs had a greater proportional abundance of functional genes belonging to carbohydrate metabolism and to sulfur metabolism than all other groups (ANOVA, Eta-squared = 0.74 and 0.61, *p* < 0.001; Tukey–Kramer, *p* < 0.05). In contrast, protein metabolism functional genes were significantly lower in relative abundance in the outer coral microbiome when compared to all other groups (ANOVA, Eta-squared = 0.61, *p* < 0.001; Tukey–Kramer, *p* < 0.01). Functional genes involved in metabolism of aromatic compounds were overrepresented in the water and coral microbiome of outer reefs when compared to the microbiome in the water and coral microbiome of inner reefs (ANOVA, Eta-squared = 0.84, *p* < 0.001; Tukey–Kramer, *p* < 0.001).

The inner coral SML microbiome was overrepresented with genes involved in phosphorus metabolism and in secondary metabolism (ANOVA, Eta-squared = 0.61 and 0.84, *p* < 0.001, Tukey–Kramer, *p* < 0.01). Functional genes within cell division and cell cycle as well as cell wall and capsule were in higher abundance in the water microbiome from inner reefs compared to the microbiome from water and corals from the outer reefs and in the microbiome from inner corals compared to the outer coral SML microbiome (ANOVA, Eta-squared = 0.79 and 0.72, *p* < 0.001; Tukey–Kramer, *p* < 0.01). Photosynthesis functional genes were overrepresented in the water microbiome of inner reefs when compared to all other groups (ANOVA, Eta-squared = 0.70, *p* < 0.001; Tukey–Kramer, *p* < 0.01).

Bacterial respiration genes were overrepresented in the microbiome of corals from both reefs when compared to the microbiome in the water column from inner and outer reefs (ANOVA, Eta-squared = 0.76, *p* < 0.001; Tukey–Kramer, *p* < 0.01). Stress response genes showed higher relative abundance in the SML microbiome of inner corals than in the water microbiome of inner reefs, and similarly more of stress response genes in the microbiome of outer corals when compared to the water microbiome from both reef zones (ANOVA, Eta-squared = 0.67, *p* < 0.001; Tukey–Kramer, *p* < 0.01). DNA metabolism genes were overrepresented in the microbiome from the water column in both reef zones when compared to the coral SML microbiome from inner and outer reefs (ANOVA, Eta-squared = 0.71, *p* < 0.001; Tukey–Kramer, *p* < 0.01).

**Fig. 4:**
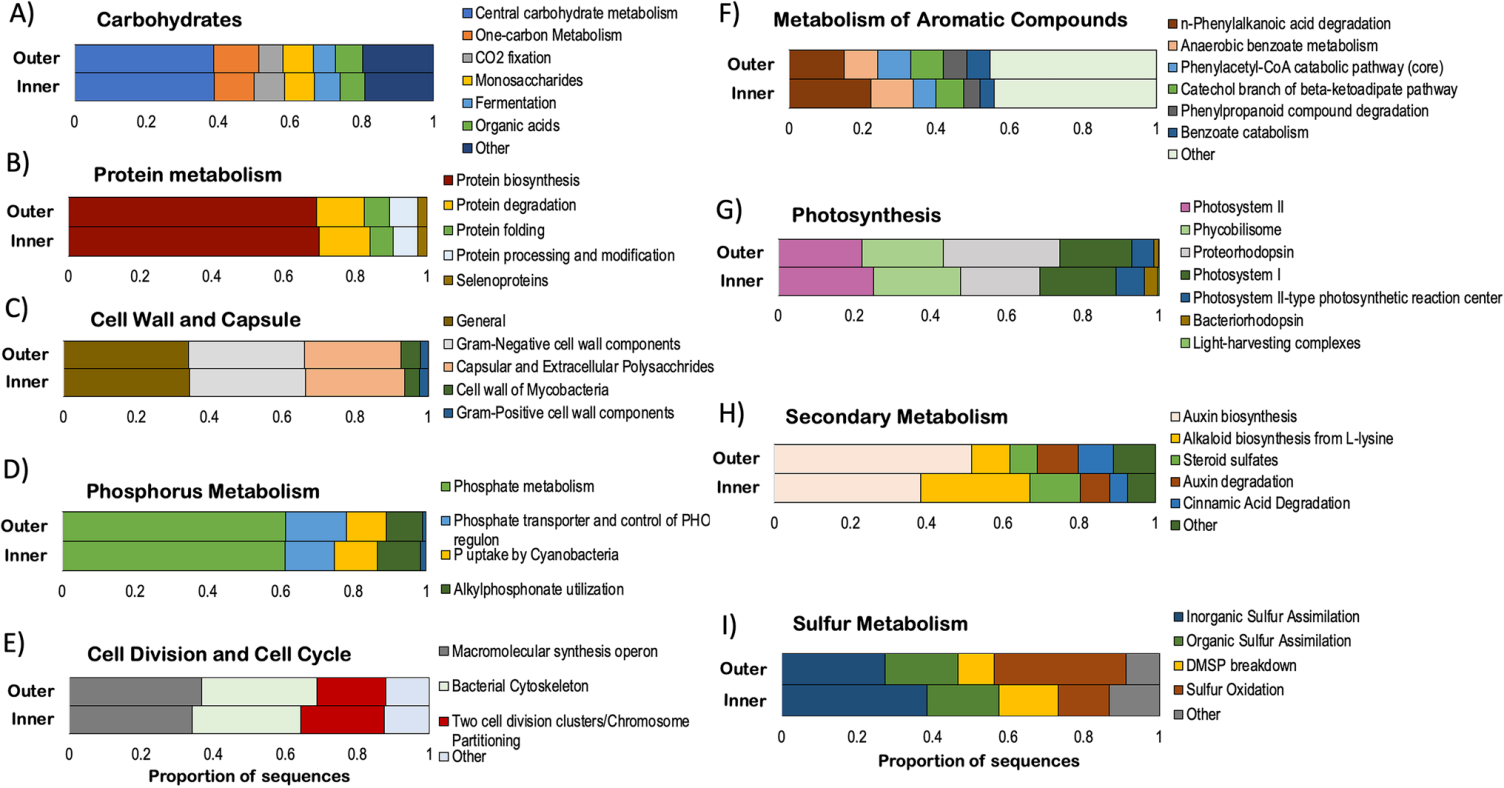
Relative abundance of bacterial functional gene subsystems (SEED subsystem 2 (**A**–**C**) and subsystem 3 (**D**–**I**)) within their respective broad functional gene category (SEED subsystem 1 the bold heading) associated with the SML microbiome of corals from inner and outer reefs

The nine broad functional gene categories (SEED subsystem level 1) that varied significantly according to the reef zone were analyzed at a higher level of resolution (SEED subsystem levels 2 and 3) to illustrate which specific functions could be under selection at reef-zone level in the coral SML microbiome only (Fig. 4). Genes involved in central carbohydrate metabolism, one-carbon metabolism, and CO_2_ fixation accounted for approximately 60% of the total carbohydrate genes both in the inner and outer coral SML metagenomes (Fig. 4A). Protein biosynthesis genes (relative abundance = 70%) dominated the protein metabolism, followed by protein degradation genes (relative abundance = 14%) (Fig. 4B). Gram negative cell wall components (relative abundance = 32%) and capsular and extracellular polysaccharides (relative abundance = 26–27%) were dominant among cell wall and capsule genes (Fig. 4C). Phosphate metabolism and transporters genes together were approximately 75% of the total phosphorus metabolism, whereas genes involved in phosphorus uptake by *Cyanobacteria* at 12% relative abundance (Fig. 4D). Within cell division and cell cycle, two cell division clusters/chromosome partitioning genes were higher in inner coral SML metagenomes (relative abundance = 23%) compared to outer coral SML metagenomes (relative abundance = 19%) (Fig. 4E). In the metabolism of aromatic compounds, n-phenylalkanoic acid degradation and anaerobic benzoate genes were more represented in inner coral metagenomes (22% in inner and 15% in outer, and 11% in inner and 9% outer, respectively), while benzoate catabolism was higher in outer coral metagenomes (6 compared to 4% in inner), and cathecol branch was approximately 8% in both groups (Fig. 4F). Proteorhodopsin genes accounted for 30% of the photosynthesis and light-harvesting complexes in outer coral metagenomes, compared to 20% in inner coral metagenomes, while photosystem II genes were lower in outer coral metagenomes (relative abundance = 22%) compared to the inner coral metagenomes (relative abundance = 25%) (Fig. 4G). In secondary metabolism, genes encoding auxin biosynthesis were higher in outer coral metagenomes than in the ones from inner reefs (relative abundances of 52% and 38%, respectively), contrasting with alkaloid biosynthesis from L-lysine genes that were more represented in inner coral metagenomes (28 versus 10%). Sulfur metabolism genes showed striking differences in proportions at subsystems level 3 (Fig. 4I), where sulfur oxidation genes were almost threefold more abundant in outer coral metagenomes than in inner coral metagenomes. Because of the differences in sulfur metabolism, in the next section, we will be focusing on the specificities of sulfur pathways and their associated taxa.

### Sulfur Metabolic Pathways in the Coral SML Microbiome

Sulfur oxidation, inorganic sulfur assimilation, and organic sulfur assimilation (including dimethylsulfoniopropionate—DMSP breakdown) were the three major sulfur subsystems in all metagenomes, accounting for approximately 90% of total sulfur metabolism genes, but the proportions of sequences related to each subsystem varied between the two reef zones. In the microbiome of outer corals, the relative abundance of sequences from each of these subsystems were evenly distributed (sulfur oxidation 33.2 ± 3.7%; inorganic sulfur assimilation 28.3 ± 2.5%; and organic sulfur assimilation 29.8 ± 1.2%). A similar pattern was detected in the water column of outer reefs (sulfur oxidation 28.5 ± 5.9%; inorganic sulfur assimilation 34.1 ± 4.1%; and organic sulfur assimilation 26.8 ± 1.7%). In contrast, in the metagenomes of inner corals, sulfur oxidation was underrepresented (12.5 ± 3.4%), when compared to inorganic sulfur assimilation (40.8 ± 6.5%) and organic sulfur assimilation (38.3 ± 1.0%). The metagenomes from the water column of inner reefs were also low in sulfur oxidation genes (15.7 ± 2.1%), and high in inorganic sulfur assimilation (38.5 ± 3.0%) and organic sulfur assimilation (34.0 ± 3.1%). Within the organic sulfur assimilation cluster, DMSP breakdown was highest in the SML microbiome of corals from inner reefs (48 ± 8.4%), followed by outer corals (33.2 ± 3.4%), inner water (31.8 ± 7.8%), and outer water (26.5 ± 7.6%). Release of dimethyl sulfide (DMS) from DMSP accounted for less than 0.001% of the sulfur metabolism genes in coral metagenomes from both reef zones.

**Fig. 5:**
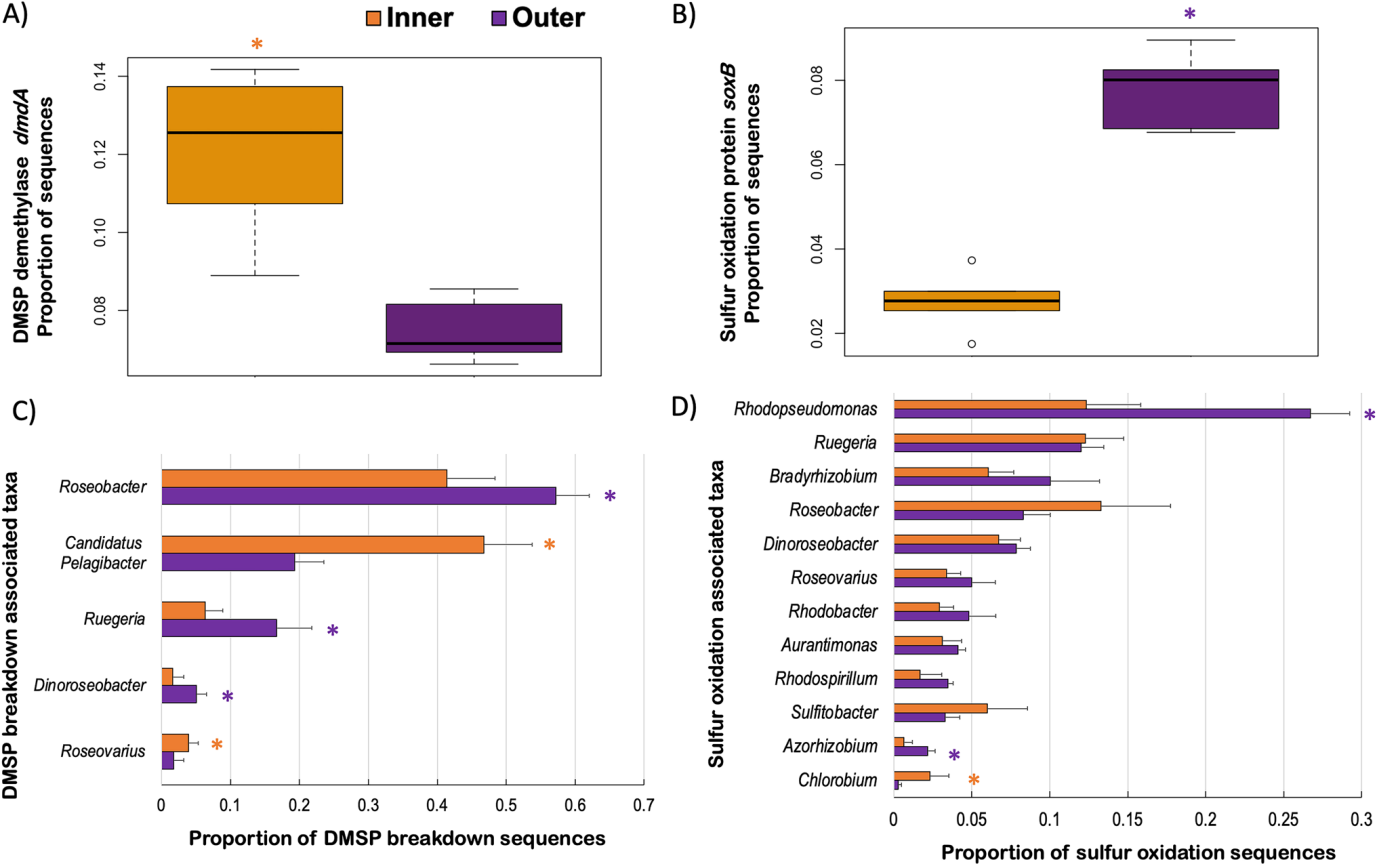
Sulfur metabolism gene pathways and respective taxa associated with the SML microbiome of *P. strigosa* from inner and outer reefs in Bermuda. Proportion of bacterial DMSP demethylase *dmdA* genes (**A**) and sulfur oxidation *soxB* genes (**B**) relative to the total sulfur metabolism genes, and of bacterial genera associated to DMSP breakdown (**C**) and to sulfur oxidation (**D**)

The proportion of sequences within the sulfur metabolism cluster encoding the enzyme DMSP demethylase *dmdA* (EC. 2.1.210) was greater in the SML microbiome of corals from inner reefs (*T*-test, *t* = 5.38, *p* = 0.001; Fig. 5A), while those encoding the sulfur oxidation protein *soxB* were higher in corals from outer reefs (*T*-test, *t* =  − 11.56, *p* < 0.001; Fig. 5B).

The bacterial genera that contributed to DMSP breakdown belonged to the same five taxa between inner and outer coral metagenomes, but these were represented in different proportions (Fig. 5C). *Roseobacter* (ANOVA, Eta-squared = 0.634, *p* < 0.001), *Ruegeria* (ANOVA, Eta-squared = 0.625, *p* < 0.001), and *Dinoroseobacter* (ANOVA, Eta-squared = 0.545, *p* < 0.001) were the main contributors to the DMSP breakdown genes in outer metagenomes, while Candidatus *Pelagibacter* (ANOVA, Eta-squared = 0.849, *p* < 0.001) and *Roseovarius* (ANOVA, Eta-squared = 0.353, *p* = 0.042) showed greater proportions in the metagenomes of inner corals. Sulfur oxidation genes were encoded by 75 genera of bacteria and the twelve most abundant taxa showed different relative abundances between inner and outer coral metagenomes (Fig. 4C). *Rhodopseudomonas* (ANOVA, Eta-squared = 0.869, *p* < 0.001) accounted for about one-quarter of all the bacterial genera encoding sulfur oxidation genes in outer coral SML, while in inner corals, the highest abundances were distributed more evenly across *Rhodopseudomonas*, *Ruegeria*, and *Roseobacter*. *Azorhizobium* (ANOVA, Eta-squared = 0.73, *p* < 0.02) was overrepresented in the sulfur oxidation genes in outer coral SML, and *Chlorobium* (ANOVA, Eta-squared = 0.63, *p* < 0.031) in the microbiome of inner corals.

## Discussion

**Fig. 6:**
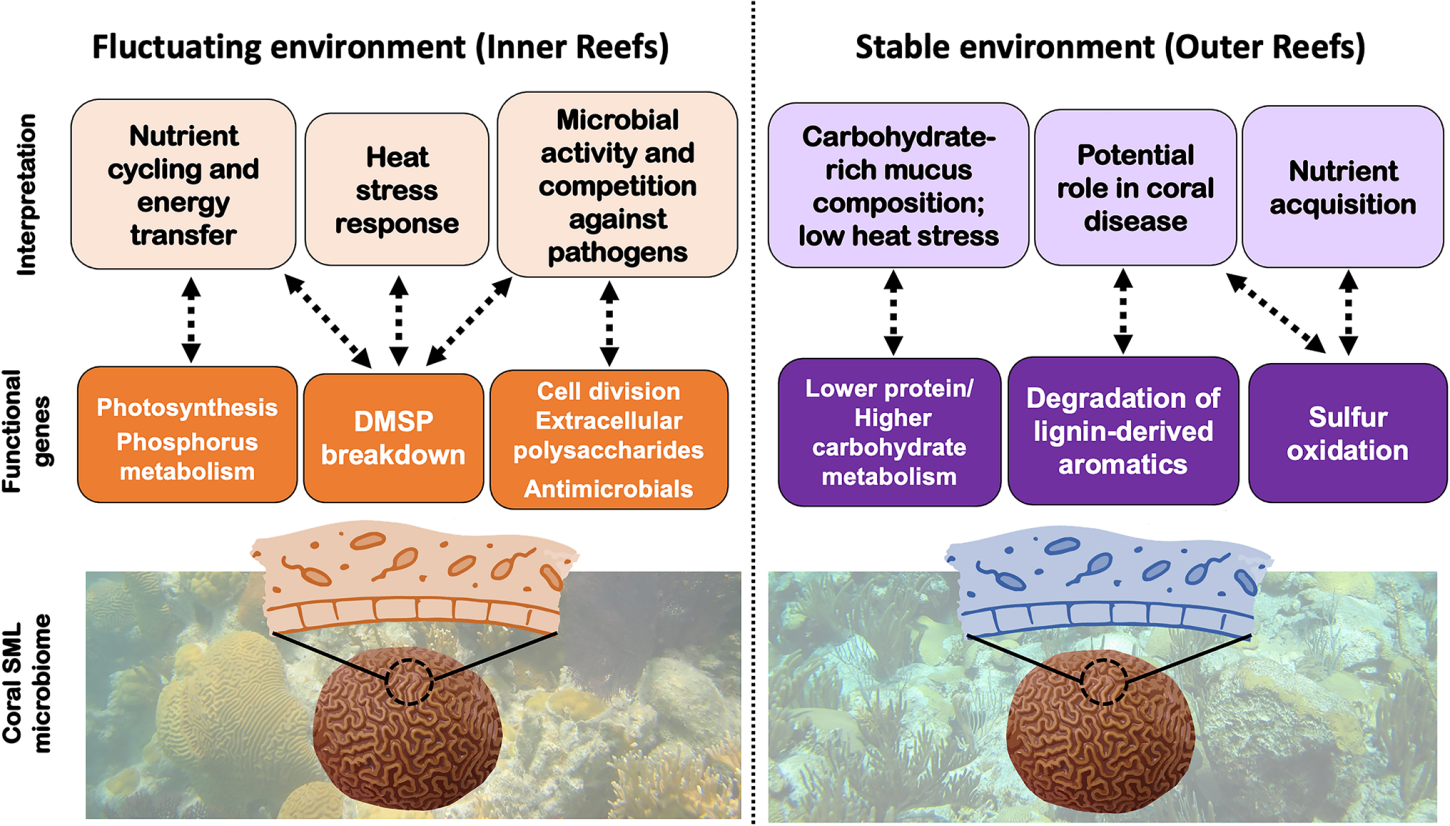
The functional metabolism of bacteria associated with the coral SML microbiome of *P. strigosa* varied across reef zones in Bermuda. In inner reefs, corals are exposed to a more fluctuating environment and their SML microbiome functional profile indicates that it provides more services related to nutrient cycling (e.g., carbon, phosphorus, sulfur), stress tolerance, and disease protection. In outer reefs, corals are exposed to a more stable environment and their SML microbiome is characterized by functional genes related to a mucus composition with a high carbohydrate to protein ratio (indicating low exposure to thermal stress), and involved in nutrient acquisition (i.e., taurine fermentation followed by thiosulfate oxidation) and coral disease (e.g., yellow-band and black-band diseases)

The metagenomes associated with the SML of *P. strigosa* and the water column from inner and outer reefs in Bermuda had similar taxonomic diversity metrics (e.g., richness, Shannon’s diversity index), corroborating that the coral SML microbiome is shaped by microbial communities in their surrounding environment [27, 28]. However, the microbial community structure (i.e., relative abundances of sequences) in Bermuda’s reef system is simultaneously selected by the coral host versus water and the local environment (i.e., inner reefs versus outer reefs), both at taxonomic and functional levels. The coral SML microbiome of *P. strigosa* was dominated by taxa commonly present in seawater that are found in other coral species [83, 84] and are selectively trapped and consumed by the coral host [19, 20]. In this study, *P. strigosa* from each reef zone had different microbial genera filling similar niches. For example, alphaproteobacterial metabolic generalists were the most abundant genera in both reef zones, represented by SAR11 Candidatus *Pelagibacter* in inner corals and SAR116 Candidatus *Puniceispirillum* in outer corals. Among phototrophs, cyanobacterium *Synechococcus* was a signature genus in inner corals and *Rhodospirillum* in outer corals. At the microbial metabolism level, the microbiome is providing key functions for coral holobiont health and ecosystem functioning; specific to each reef zone (Fig. 6).

### The Coral SML Microbiome from a Fluctuating Environment Provides More Services Related to Nutrient Cycling, Stress Tolerance, and Disease Protection

The coral and water microbiomes from inner reefs reflect a highly productive and fluctuating system when compared to outer reefs. The overrepresentation of photosynthetic bacteria in the water column and the coral SML of inner reefs mirrored the elevated abundance of functional genes related to photosynthesis and phosphorus metabolism. *Synechococcus* is a main primary producer in the picoplankton, reaching the highest concentrations off Bermuda during the spring bloom [85], the same season as this study. *Synechococcus* was highly abundant in the metagenomes and MAGs from inner reef corals and, therefore, could be the main contributor to photosynthesis and phosphorus metabolism genes. We used metagenomics to describe the abundance of genes in the microbiome which identifies functional genes that are important for the microbes in that environment [65, 66] although it does not measure which functional genes are being expressed at the point the sample was taken. In the coral SML metagenomes, we identified that phosphorus metabolism was mostly comprised of genes involved in phosphate metabolism and phosphorus uptake by *Cyanobacteria* (e.g., *Synechococcus*). The coral SML is rich in phosphate when compared to the water column [17], contributing to primary productivity in benthic and pelagic reef ecosystems [86]. The coral SML efficiently traps *Synechococcus* from the pelagic picoplankton, which contributes to the flux of particulate organic matter (POM) from the water column to benthos [18]. Corals selectively remove *Synechococcus* and other pelagic microbes via feeding, and promote the growth of diverse picoplankton, shaping the microbial community in the surrounding reef water [19, 20]. Heat-stressed corals preferentially fed on *Synechococcus* to access the high nitrogen content in their cells and to compensate for the loss of nitrogen from algal endosymbiont *Symbiodiniaceae* during recovery from bleaching [87]. The inner lagoon patch reefs in Bermuda are exposed to greater environmental fluctuations, particularly changes in temperature [48–50, 54]. Therefore, the high abundance of *Synechococcus* in the water column and in the SML of *P. strigosa* could be contributing to the energy transfer from pelagic to benthic trophic levels, and to the coral thermal tolerance in the inner lagoon reefs of Bermuda.

Microbial activity, growth, and competition are higher in the inner reefs than in the outer reefs in Bermuda, as suggested by the functional profiles from the coral SML and water column. Functional genes related to cell division and cell cycle, such as those encoding two cell division and chromosome partitioning, are in greater abundance in inner coral metagenomes. In addition, there is a high relative abundance of cell wall and capsule functional genes, including those encoding capsular and extracellular polysaccharides in the microbial communities of inner reefs. Microbial extracellular polymeric substances (EPS) play a crucial role in marine environments, increasing dissolved organic carbon (DOC) levels, binding and removing heavy metals from the water column, and influencing oxygen levels [88]. Microbial growth rates in the coral SML are higher under elevated DOC levels [89]; therefore, the abundance of genes related to EPS suggests an increased microbial activity in the SML of corals from inner reefs. DOC levels are also associated with larger quantities of exudates released by benthic macroalgae in coral reefs [90]. Even though both reef zones showed similar coral cover; turf and macroalgae were more abundant in inner reefs (Figure S1), indicating that the DOC levels induced by macroalgae exudates could be higher in this reef zone in Bermuda. The microbial communities associated with inner corals are enriched with genes belonging to secondary metabolism, including a high relative abundance of genes encoding alkaloid biosynthesis from L-lysine. *Cyanobacteria* are key producers of marine alkaloids [91], which could be contributing to the high levels of these functional genes in coral metagenomes from inner reefs. Alkaloids function as antimicrobials [92, 93]; therefore, the overrepresentation of alkaloid biosynthesis genes indicates greater microbe-microbe competition in the coral SML microbiome from inner reefs. Microbial competition and production of antimicrobial compounds offer protection against opportunistic pathogens to the coral host [93–96] and thus promoting a more beneficial SML microbiome on *P. strigosa* colonies inhabiting inner reefs compared to outer reefs.

Dimethylsulfoniopropionate (DMSP) breakdown genes (e.g., *dmdA*) belong to the organic sulfur assimilation subsystem and were more abundant in the SML microbiome of inner corals across all metagenomes. DMSP is a valuable component in marine environments, with high turnover rates, and is an important link between primary production and bacterial activity [97]. *Pelagibacter ubique*, for example, exclusively assimilates sulfur from organic sources such as DMSP [98], and was a key taxon associated with DMSP breakdown in inner reefs. The coral metagenomes had greater proportions of *Pelagibacter* than the water metagenomes suggesting that the coral SML is providing a DMSP-rich environment for bacterial growth. DMSP is considered an antioxidant [99, 100], and increased levels of this compound have been associated with stress response in the coral holobiont [32, 101, 102]. DMSP that reaches the coral SML is produced by the coral-algal symbiont [103] and the coral animal, especially under thermal stress [4, 104]. Bacteria subsequently use this compound as a sulfur and carbon source, relying on the *dmdA* gene to encode DMSP methyltransferase to incorporate sulfur to amino acids (e.g., methionine) [98, 105]. Sulfur as a product of DMSP breakdown can also be used by bacteria to form sulfur-based antimicrobial compounds such as tropodithietic acid (TDA), which protects the coral host by inhibiting the growth of pathogens [26]. Therefore, DMSP breakdown is considered one of the main beneficial services provided by the coral microbiome to the holobiont, because it is linked both to disease protection and nutrient cycling [30]. The sulfur metabolism of the microbiome of inner corals, which prioritizes sulfur assimilation and DMSP breakdown, is another indicator that the coral holobiont from inner reefs is responding to a more fluctuating thermal environment and potentially is associating with a microbiome that is more beneficial for this environment.

### The Coral SML Microbiome from a Stable Environment Indicates Less Exposure To Stress, But Is Potentially Under Nutrient Limitation and More Prone to Coral Disease

The microbial functional profile in outer reefs was characterized by a carbohydrate-dominated metabolism, and a reduction in protein metabolism genes and is indicative of the variation of the SML composition between corals from the two reef zones. Corals secrete a polysaccharide protein lipid complex that is colonized by an abundant microbial community [14]. The proportions of carbohydrates, proteins, and lipids in the coral mucus vary according to factors such as coral species [106–108], stress [109], and reef environments [108]. The coral SML microbiome is strongly shaped by the mucus composition [110]; therefore, the high relative abundance of microbial genes involved in carbohydrate metabolism and the loss of protein metabolism genes is consistent with corals from outer reefs producing mucus with a higher carbohydrate to protein ratio. Heat-stressed corals had an increase in protein content, and higher microbial activity, compared to healthy corals under mild temperature conditions [111]. Corals from the outer reefs in Bermuda are less exposed to thermal fluctuations [49, 50, 112] and the microbial community structure from their mucus can be modeled according to their local thermal environment [54]. The reduction in protein metabolism genes and overrepresentation of carbohydrate metabolism genes suggest that the mucus composition of corals from outer reefs is characteristic of corals under low exposure to thermal stress.

Metabolism of aromatic compounds was a signature function both in the coral and water microbiomes from outer reefs. The gene encoding the enzyme muconate cycloisomerase (EC 5.5.1.1) is part of the catechol branch of beta-ketoadipate pathway and was found at lower relative abundance in the SML microbiome of inner corals (1%), than in outer corals (8%). The beta-ketoadipate pathway is commonly present in soil microbes, involved in the degradation of lignin-derived aromatics such as cathecol to citric acid cycle intermediates [113], although lignin degradation genes are found in many marine bacterial strains of *Pseudoalteromonas*, *Marinomonas*, and *Thalassospira*, among others [114]. The sources of lignin that is being degraded by the microbiome of outer reefs are unresolved, as this compound is characteristic of vascular land plants, but lignin has been described to be within the cells of one marine macroalga species, *Calliarthron cheilosporioides* [115]. Interestingly, an increased relative abundance of genes responsible for lignin degradation in the coral mucus microbiome was associated to yellow-band disease and attributed to lysing of the coral tissue [116]. Therefore, the role of lignin degradation in the coral microbiome could be related to coral health and needs to be further investigated.

Outer reef corals showed a higher abundance of total sulfur metabolism genes in their SML microbiome when compared to the microbiome of inner corals. An increase in the relative abundance of sulfur metabolism genes in the coral microbiome has been associated with low pH, thermal stress [25], and bleaching [117]. However, the colonies were visually healthy, and the environmental conditions were mild during sampling collection (Table S2). The microbiomes of outer and inner corals adopted different sulfur metabolism strategies according to their local environment. Sulfur oxidation was overrepresented in the outer water and coral metagenomes, in comparison to metagenomes from inner reefs, which invested more in inorganic and organic sulfur assimilation. Sulfur oxidation in the coral microbiome is much less understood than sulfur assimilation and is usually studied in the context of black-band disease (BBD). BBD is one of the most virulent and widespread of all coral diseases and develops as a polymicrobial consortium dominated by cyanobacteria, sulfur-reducing and sulfur-oxidizing bacteria (SRB and SOB, respectively) that change in relative abundance across stages of infection [118, 119]. The disease manifests as a dark microbial mat between living tissue and exposed skeleton resulting from tissue necrosis with fast progression rates [120]. BBD prevalence in *P. strigosa* colonies from outer reefs was the highest across Bermuda reef zones and among other coral host species, despite the pristine water quality and marine protected area status [121]. Sulfur oxidation genes from *Rhodobacteraceae* were proportionally higher in outer coral metagenomes and were identified in BBD lesions [122]. However, SOB do not seem to be directly linked to BBD pathogenicity, but likely function as secondary colonizers [122]. The high sulfide concentrations created by SRB and loss of oxidizers within the BBD mat are linked to coral tissue degeneration [123, 124]. Sulfur oxidation in outer reef corals could be part of a healthy coral microbiome metabolism, related to amino acid degradation as a sulfur source to bacteria. The *soxB* gene pathway is part of the Sox enzyme complex that allows a phylogenetically diverse group of SOB to convert thiosulfate to sulfate [125] and was significantly more abundant in the outer coral SML microbiome. Thiosulfate can be a fermentation product of taurine [126]. Taurine dioxygenases were present in MAGs associated with the coral microbiome, suggesting that the microbes are using this amino acid as a nutrient source [47], especially in more oligotrophic waters such as in the outer reefs of Bermuda. The role of sulfur metabolism in coral health and disease susceptibility needs to be further studied, and the Bermuda reefs provide a natural laboratory system for coral microbiome research.

### The Coral SML Microbiome Has Distinct Features from the Water Column Microbiome Independent of Local Reef Zone

The coral SML microbiome of *P. strigosa* from inner and outer reefs shared some taxonomic and functional features, despite the strong effect caused by the local reef zone. *Pseudomonas* was the only genus that was overrepresented in the coral SML from both reef zones in comparison to their local water microbiome. *Pseudomonas stutzeri* was identified by our MAGs particularly in outer reef samples. Marine strains of *P. stutzeri* have been isolated from the water column and sediment, and their major ecological roles are related to denitrification and sulfur oxidation [127]. *P. stutzeri* could be playing an important nutrient cycling role in the coral SML and this relationship requires further investigation. At functional level, the coral SML microbiome showed greater proportions of respiration and stress response genes, independent of their local reef zone. The coral microbiome was dominated by heterotrophs that take advantage of the rich carbon sources in the mucus, therefore, increasing microbial respiration, i.e., oxygen consumption, when compared to the free-living, photosynthetic, and oxygen-producing microbial community in the surrounding water [25, 37]. A greater relative abundance of pathways associated with stress response may indicate passive or active selection within the holobiont, which could be a source of resilience according to the hologenome theory of evolution, if these microbial genes can be vertically transmitted [128]. This potential selection of microbial stress response genes relates to the MMTA theory that is yet to be corroborated and assumes that the coral holobiont benefits from inheritable microbial taxa and/or genes acquired and/or selected in the coral microbiome when exposed to environmental changes [29]. Future research should investigate whether the coral holobiont is selecting microbial genes differently in response to environmental stress and whether they are passed on through generations.

## Conclusion

Coral health has sharply decreased in the last two decades as coral bleaching and disease outbreaks have become more frequent worldwide, particularly correlated to rising seawater temperature [129–132]. Conservation efforts to improve coral health by promoting or maintaining a beneficial microbiome (e.g., development of probiotics) depend on a detailed understanding of the dynamics of microbial taxa and functional profiles [36, 133, 134].

Our results showed specific coral-microbial gene functions and taxa that are being selected, either passively or actively, according to the local environment, in response to primary productivity, stress, and nutrient cycles, particularly the sulfur cycle. The fluctuating environment in the inner patch reefs of Bermuda could be driving a more beneficial coral SML microbiome for the prevailing environment via local long-term acclimatization, potentially increasing holobiont resistance to thermal stress and disease. This reef zone could be a source of a coral holobiont that is more resilient to environmental changes in comparison to outer reefs. Coral restoration programs, especially when using transplantation of coral colonies across different areas of the reef, should design strategies that consider the trade-offs involving coral microbiome acclimatization at reef scale.

## Supplementary Information

Below is the link to the electronic supplementary material.

Supplementary file1 (DOCX 3747 KB)

## Data Availability

The metagenomic data from this study is publicly available in the SRA database as BioProject PRJNA595374 (https://www.ncbi.nlm.nih.gov/bioproject/595374) and in MG-RAST as public study SDSU_BIOS_2017 (mgp81589; https://www.mg-rast.org/linkin.cgi?project=mgp81589).
