# Supplementary material for: Coral and Seawater Metagenomes Reveal Key Microbial Functions to Coral Health and Ecosystem Functioning Shaped at Reef Scale
Source: Microb Ecol. 2022 Aug 15;86(1):392–407. doi: 10.1007/s00248-022-02094-6 (PMC10293411; doi:10.1007/s00248-022-02094-6)
Supplement: Supplementary file 1 — Supplementary file1 (DOCX 3747 KB) [file 248_2022_2094_MOESM1_ESM.docx]

**Supplemental Material**

Table S1. Metagenomic sequences coverage and annotation hits through MG-RAST (as of April 8^th^, 2021).

| Metagenome name | Sample ID | Total number of sequences | Bacterial sequence hits | Archaeal sequence hits |
| --- | --- | --- | --- | --- |
| Inner_Reef_1_AA | mgs602127 | 932,522 | 409,892 | 2,090 |
| Inner_Reef_1_LL | mgs602130 | 1,264,982 | 539,254 | 3,004 |
| Inner_Reef_1_water_1 | mgs602169 | 860,221 | 522,241 | 1,809 |
| Inner_Reef_1_water_2 | mgs602172 | 780,980 | 556,559 | 1,823 |
| Inner_Reef_2_AA | mgs602133 | 1,115,369 | 473,425 | 2,116 |
| Inner_Reef_2_water_1 | mgs602175 | 897,812 | 594,281 | 2,055 |
| Inner_Reef_2_water_2 | mgs602178 | 968,692 | 746,806 | 2,501 |
| Inner_Reef_3_AA2 | mgs602145 | 626,624 | 262,136 | 1,522 |
| Inner_Reef_3_LL1 | mgs602142 | 898,828 | 354,127 | 1,543 |
| Inner_Reef_3_LL2 | mgs602148 | 870,627 | 343,187 | 2,212 |
| Inner_Reef_3_water_1 | mgs602181 | 642,681 | 478,392 | 1,768 |
| Inner_Reef_3_water_2 | mgs602184 | 752,643 | 584,613 | 1,604 |
| Outer_Reef_1_AA | mgs602151 | 921,996 | 341,979 | 2,208 |
| Outer_Reef_1_LL | mgs602154 | 864,577 | 609,245 | 3,968 |
| Outer_Reef_1_water_1 | mgs602187 | 985,985 | 610,602 | 2,575 |
| Outer_Reef_1_water_2 | mgs602190 | 1,221,790 | 705,749 | 2,735 |
| Outer_Reef_2_AA | mgs602157 | 858,085 | 548,099 | 3,710 |
| Outer_Reef_2_LL | mgs602160 | 1,368,678 | 906,029 | 5,470 |
| Outer_Reef_2_water_1 | mgs602193 | 1,025,086 | 759,119 | 4,161 |
| Outer_Reef_2_water_2 | mgs602196 | 526,746 | 455,237 | 3,295 |
| Outer_Reef_3_AA | mgs602163 | 646,510 | 287,792 | 1,631 |
| Outer_Reef_3_LL | mgs602166 | 684,623 | 486,791 | 3,102 |
| Outer_Reef_3_water_1 | mgs602199 | 421,976 | 250,062 | 1,020 |
| Outer_Reef_3_water_2 | mgs602202 | 657,501 | 424,819 | 1,960 |

Table S2. Environmental parameters (mean ± SD) in the water column (4 - 5m depth) of inner and outer reefs of Bermuda.

| Reef Zone | Temperature (˚C) | pH | Chlorophyll-a concentration (µg/L) | Dissolved Oxygen (mg/L) | Dissolved Oxygen Saturation (%) |
| --- | --- | --- | --- | --- | --- |
| Inner Reefs | 23.83 ± 0.21 | 8.27 ± 0.03 | 1.79 ± 0.23 | 7.22 ± 0.04 | 106.53 ± 0.53 |
| Outer Reefs | 23.15 ± 0.33 | 8.27 ± 0.03 | 1.32 ± 0.06 | 7.30 ± 0.34 | 106.53 ± 5.44 |


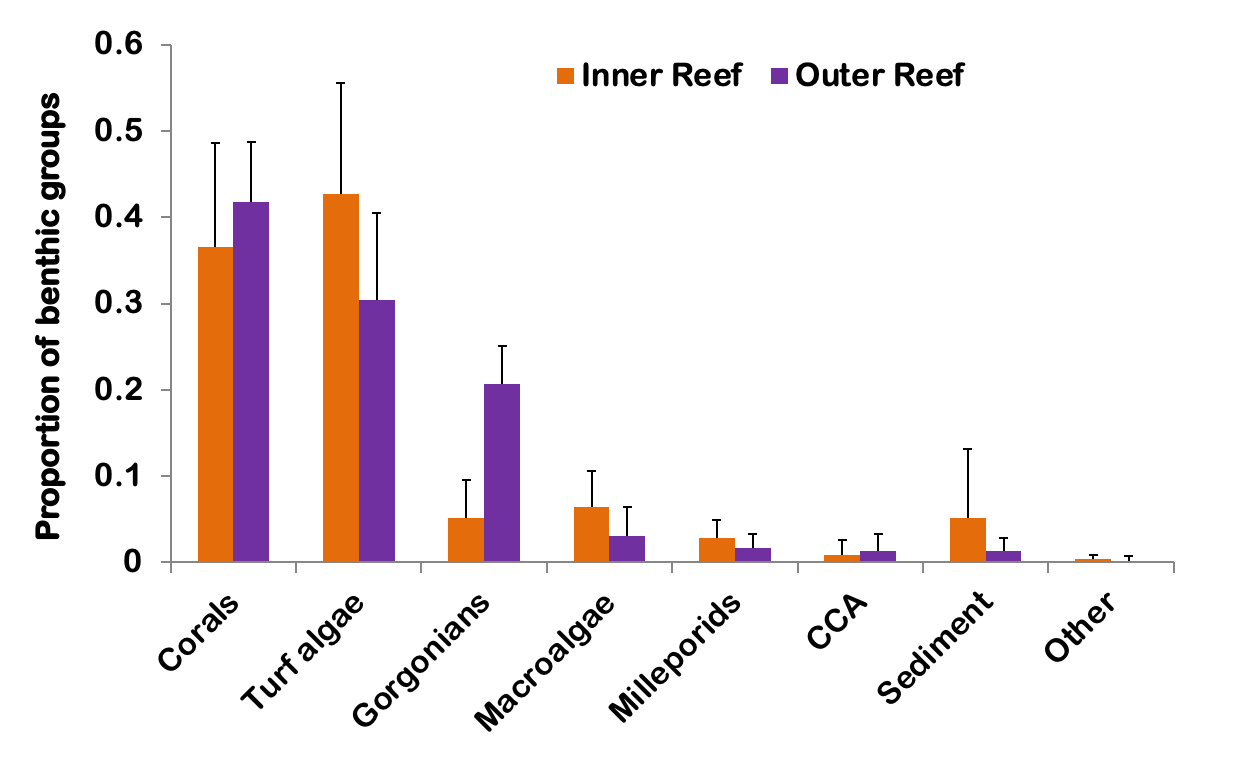


Figure S1. Benthic coverage (mean ± SD) of inner and outer reefs of Bermuda.
